# Evaluation of the anti-oxidant effect of ascorbic acid on apoptosis and proliferation of germinal epithelium cells of rat testis following malathion-induced toxicity

**DOI:** 10.22038/ijbms.2020.35952.8608

**Published:** 2020-05

**Authors:** Faezeh Ghorbani-Taherdehi, Mohammad Reza Nikravesh, Mehdi Jalali, Alireza Fazel, Mahmoud Gorji-Valokola

**Affiliations:** 1 Department of Anatomy and Cell Biology, Mashhad University of Medical Sciences, Mashhad, Iran; 2 Microanatomy Research Center, Mashhad University of Medical Sciences, Mashhad, Iran; 3 Department of Pharmacodynamics and Toxicology, School of Pharmacy, Mashhad University of Medical Sciences, Mashhad, Iran

**Keywords:** Apoptosis, Ascorbic acid, Malathion, PCNA, Rat, TUNEL technique

## Abstract

**Objective(s):**

The aim of this study was to determine the protective role of ascorbic acid on apoptosis and proliferation of spermatogonia and primary spermatocyte cells after malathion administration as an organophosphate pesticide in rat testis.

**Materials and Methods:**

Thirty male Wistar rats were randomly divided into five groups of 6 rats each, including control (no intervention), sham (normal saline 0.09%), malathion (50 mg/kg), malathion plus ascorbic acid (50 mg/kg and 200 mg/kg, respectively), and ascorbic acid (200 mg/kg) groups. Malathion and ascorbic acid were administrated via intraperitoneal injection once per day and seven times per week. After 6 weeks, animals were sacrificed, and testis tissue was used for evaluation of apoptosis and proliferation of germinal epithelium cells using the TUNEL and PCNA staining techniques.

**Results:**

The results of TUNEL staining showed that the numbers of apoptotic cells in spermatogonia and primary spermatocyte cells were significantly increased in the malathion 50 mg/kg group vs control group (*P*<0.001). Co-administration of malathion 50 mg/kg and ascorbic acid 200 mg/kg significantly decreased the apoptotic cells in both cell types in comparison with malathion 50 mg/kg group (*P*<0.001). The results of PCNA staining revealed that the proliferation of these cells was significantly decreased in malathion 50 mg/kg group vs control group (*P*<0.001), and malathion 50 mg/kg plus ascorbic acid 200 mg/kg administration increased the proliferation of cells compared with malathion 50 mg/kg group (*P*<0.001).

**Conclusion:**

The results provide evidence that ascorbic acid showed preventive effects on malathion-induced toxicity in male rat testis.

## Introduction

Malathion (Diethyl methoxy thiophosphoric tiosccinate), an organophosphate (OP) pesticide, is a broad and selective insecticide that is widely used to control insects and carriers of diseases in many countries. This OP pesticide is absorbed through the skin, mucous membranes, gastrointestinal tract, and respiratory tract (1). Malathion is more unstable than other OP pesticides and is rapidly degraded by hydrolysis, photolysis, and biodegradation (2). Malathion can affect various organs of the body, including the liver, kidneys, pancreas, immune system, genitourinary system, etc. (3). The main mechanism of malathion poisoning is the irreversible inhibition of the acetylcholinesterase enzyme (4). Malathion is metabolized to malaoxon, the toxic metabolite of malathion, through the cytochrome P450 enzyme in the liver, which has a higher toxicity than malathion (5-7). The most important mechanism of OP toxicity is the production of free radicals. In the body, between the production and removal of free radicals is a balance under normal conditions, while the imbalance in this process causes oxidative stress and apoptosis (8-10).

Apoptosis, a planned death and an energy-dependent process, is a physiological process that controls the number of cells in the tissues and organs, regulates cellular differentiation, immune responses, etc., which is activated by intracellular and extracellular factors (11). Apoptosis and cell proliferation are important processes in the normal function of various cells (12). Apoptosis in the process of natural spermatogenesis also plays an important role by decreasing germ cells, eliminating abnormal sperms, aging, and immature and damaged cells (12). OP pesticides can cause apoptosis from several pathways (13). Malathion, as a known OP pesticide at the age of puberty can create changes in semen parameters and survival, movement, and morphology of sperm (14, 15), which affect the final stages of maturation of the spermatogenesis cells. These effects result in DNA damage and reduce and change the structure of chromatin in spermatogonia and spermatid cells (16). Cell proliferation is essential in the process of restoring and surviving multi-cell organisms. The proliferation cell nuclear antigen (PCNA) is an essential nuclear protein for the metabolism of nucleic acid, which is necessary for multiple cell cycles, including replication and repair of DNA (17). PCNA is also involved in the control of the cell cycle through interaction with the cycloline cdk complex (18-21). PCNA expression has a direct relationship with mitotic activity. The spermatogonia and primary spermatocyte cells in the seminiferous tubules permanently are mitotic and can be used as markers for proliferation cells (22, 23).

Antioxidants are a variety of compounds that act in both enzymatic and non-enzymatic ways to prevent excessive production of free radicals (ROS) and the damage caused by them (24). Ascorbic acid (AA/L-ascorbate or vitamin C) belongs to the family of non-enzymatic antioxidants. The physicochemical properties of ascorbic acid are solid white color, low molecular weight, and water-solubility. Ascorbic acid also is an electron carrier in chemical reactions (25, 26), which is essential for many processes in the body, including bone formation, scar repair (27), and reducing free radical production, oxidative stress, and lipid peroxidation (28).

Therefore, in this study, the protective effect of ascorbic acid was evaluated on the apoptosis and proliferation of spermatogonia and primary spermatocyte cell-induced malathion in the rat testes.

## Materials and Methods

***Animals and treatment***

In this experiment, 30 adult male Wistar rats (two-months old), with a weight range of 200–250 g, were randomly divided into five groups and each group consisted of 6 rats (29-31), including control (no intervention), sham (normal saline 50 mg/kg), malathion 50 mg/kg (32-34), malathion 50 mg/kg plus ascorbic acid 200 mg/kg, and ascorbic acid 200 mg/kg groups (35). Malathion, solvent (normal saline), and ascorbic acid were injected intraperitoneally (IP) (28, 36). After 6 weeks (37), the rats were anesthetized by IP injection with ketamine/xylazine (60 mg/kg and 6 mg/kg, respectively). Then, rats were sacrificed, and the testes were used for evaluation of apoptosis and cell proliferation by PCNA and terminal deoxynucleotidyl transferase dUTP nick end labeling (TUNEL) techniques, respectively. Rats were kept in standard conditions (temperature 23±2 ^°^C and light/dark cycle of 12 hr) and had free access to food and water during the experiment. Study was not also blind. All the experimental protocols were approved by the Ethical Committee of Mashhad University of Medical Science (IR.MUMS.REC.1393.136) and this experiment was conducted at the Department of Anatomy and Cell Biology, School of Medicine, Mashhad University of Medical Sciences, in 2015. 

***Chemicals***

In this study, technical malathion 99% was purchased from Kavosh Kimia Kerman Company, and the stock solution with a concentration of 50 mg/ml was freshly prepared in normal saline (0.9% NaCl w/w). Ascorbic acid from Sigma–Aldrich (St. Louis, Missouri, USA), kit PCNA from Zymed Company (Germany), and kit TUNEL from Roche Company (Germany) were obtained. All other chemicals used in this study were of the highest purity available and purchased from Sigma–Aldrich (Germany).

***Histological analysis***

The PCNA and TUNEL immunohistochemical techniques were done according to the method of Sargazi *et al*. 2015 (38). In summary, the steps included sampling, fixing, dehydration, clearing, saturating, and tissue molding. After removing the testes from the rat’s body, they were immediately fixed in paraformaldehyde 4%, solved in 100 ml phosphate buffer saline (PBS) for 14-16 hr and dehydrated in ascending grades of alcohol (70%–100%) for 2 hr. Then, tissues were cleared in alcohol-xylene to 50:50 ratio, in xylene 3 times, and fixed in paraffin. Next, they were cut in 5 μm slices with a microtome and placed on poly L-lysine slides. Slides were deparaffinized and hydrated in descending grades of ethanol. Finally, tissues were analyzed through the PCNA and TUNEL immunohistochemical techniques and observed with an optical microscope (38).

***TUNEL immunohistochemical technique***

The TUNEL peroxidase kit was used for evaluation of apoptosis in testis tissues (*in situ* cell death detection Kit- POD, Roche, Germany) (38). In summary, the slices were deparaffinized, hydrated in descending grades of alcohol, and next, with 20 mg/ml K protein kinase incubated for 20 min at room temperature. Then, the slices were incubated with a reactive TUNEL mixture consisting of 450 μl terminal deoxynucleotidyl transferase enzyme solution and 50 μl label solution for 1 hr, at 37 ^°^C. Then, Deoxyuridine Triphosphate (dUTP) conjugated by dioxygenproxidase was added and the slides were covered with a lid. Next, dioxygen and hydrogen peroxide (Converter-POD) were added to the samples for 30 min at 37 ^°^C and diaminobenzidine (DAB) (DAB powder 6 mg, PBS 10 ml, H_2_O_2_ 3% 10 μl) was added to samples. Finally, hematoxylin staining was used and the apoptotic cells emerged in brown color (39, 40).

***PCNA immunohistochemical technique***

The PCNA kit (Zymed Co., USA) was used for evaluation of proliferation of testis cells by modified PCNA staining (41, 42). In summary, samples were deparaffinized and hydrated with reduced grades of ethanol (70%–95%). The specimens were washed thrice with PBS, and then the activity of intra-tissue peroxidase was blocked by 100 μl mixture of 3% H_2_O_2_ in methanol for 15 min at room temperature. Samples were incubated with primary antibody (mouse monoclonal PCNA concentrate, dilution 1:100, clone PC 10 BIOCARE) at room temperature for 30–60 min and then incubated with Streptovidine peroxidase and Diaminobenzidine chromogen (DAKO) (DAB powder 6 mg, PBS 10 ml, H_2_O_2_ 3% 10 μl) for 10 min. Finally, the staining was performed with Arsine Blue and positive PCNA cells were identified with darker cores and brown color (20).

***Stereology technique***

*Quantification of apoptotic cells*

Five to ten testis sections were scanned and imaged by a light microscope (Olympus X 51). The images were transferred to a computer using a high-resolution camera (BX51, Japan). Morphometrical techniques were used to calculate TUNEL positive spermatogonia and primary spermatocyte cells per unit area in the testis. The numbers of TUNEL positive cells were calculated by grades Unbiased frames and Image J program. 

The mean number of TUNEL positive cells per unit area (NA) in different types of spermatogonia and primary spermatocyte cells in different groups of rats was evaluated using the following formula: 

NA=∑Q/a/f∑p (43).

In this formula, “ΣQ” is the sum of counted particles appearing in sections, “a/f” isthe area associated with each frame, and “ΣP” is the sum of frame associated points hitting space (43).

***Statistical analysis***

In this experimental, data were analyzed using the SPSS software package (ver. 16, Chicago, IL, USA). Immunohistochemical results are expressed as mean ± SD. Statistical analysis was done using One-Way ANOVA and Tukey-Kramer tests in order to compare the mean differences between the groups, and the differences were considered statistically significant at *P*<0.05.

## Results

***Evaluation of malathion-induced apoptosis and protective effect of ascorbic acid in spermatogonia cells by TUNEL technique***

**Figure 1:**
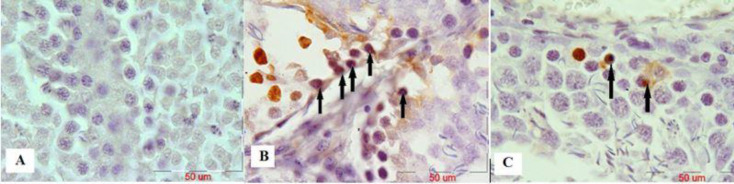
The microscopic sections of spermatogonia cells in male rat testis by the TUNEL technique. Sections were cut into 5 µm thickness and stained with Hematoxylin. TUNEL-positive nuclei are seen in brown. Apoptotic cells are indicated by arrows. Control group, no TUNEL-positive cells, magnification ×100 (A), Mal 50 mg/kg group showed increased number of TUNEL-positive cells compared with the control group, magnification ×100 (B), and malathion 50 mg/kg along with ascorbic acid 200 mg/kg group showed significantly decreased number of TUNEL-positive cells compared with the malathion 50 mg/kg group, magnification ×100 (C)

**Figure 2:**
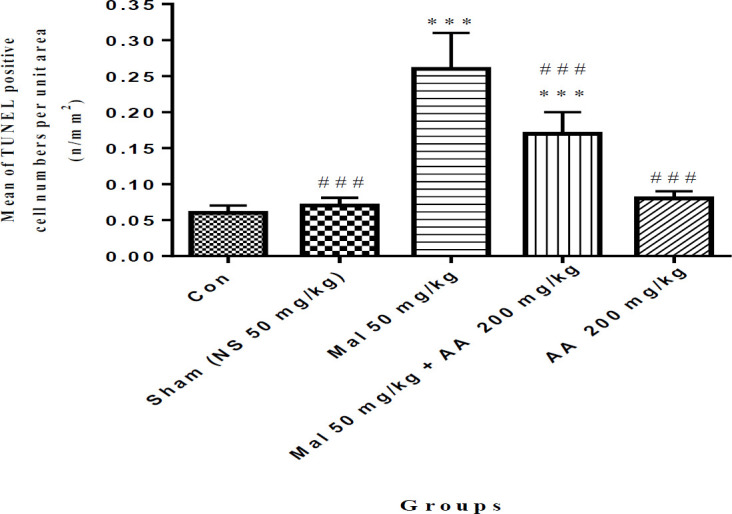
Comparison of the number of apoptotic cells in spermatogonia cells among different groups, data are shown as mean±SD, One-way ANOVA, and Tukey-Kramer. Mal 50 mg/kg and AA 200 mg/kg were administered IP once a day and seven times per week. ***P<0.001 compared to the Con group, and ###*P*<0.001 compared to Mal group

The results of the histological study showed that the number of apoptosis cells in spermatogonia cells was significantly increased in the malathion 50 mg/kg group compared with the control group (*P*<0.001). Co-administration of malathion 50 mg/kg and ascorbic acid 200 mg/kg significantly decreased the number of apoptotic cells compared with malathion 50 mg/kg (*P*<0.001). A significant reduction in the number of apoptotic cells in spermatogonia cells was observed in ascorbic acid 200 mg/kg group (*P*<0.001) (Figures 1 and 2). However, the number of apoptotic cells was not significantly different in sham, ascorbic acid, and control groups (*P*>0.05).

***Evaluation of malathion-induced apoptosis and protective effect of ascorbic acid on primary spermatocyte cells by the TUNEL technique***

**Figure 3:**
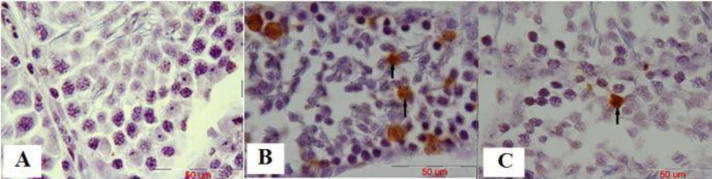
The microscopic sections of primary spermatocyte cells in male testis rat by the TUNEL technique. Sections were cut into 5 µm thickness and stained with Hematoxylin. TUNEL-positive nuclei are seen in brown. Apoptotic cells are indicated by arrows. Control group, no TUNEL-positive cells, magnification ×100 (A). In malathion 50 mg/kg group the number of TUNEL-positive cells increased compared with the control group, magnification ×100 (B), and in malathion 50 mg/kg along with ascorbic acid 200 mg/kg group the number of TUNEL-positive cells decreased significantly compared with the malathion 50 mg/kg group, magnification ×100 (C)

**Figure 4:**
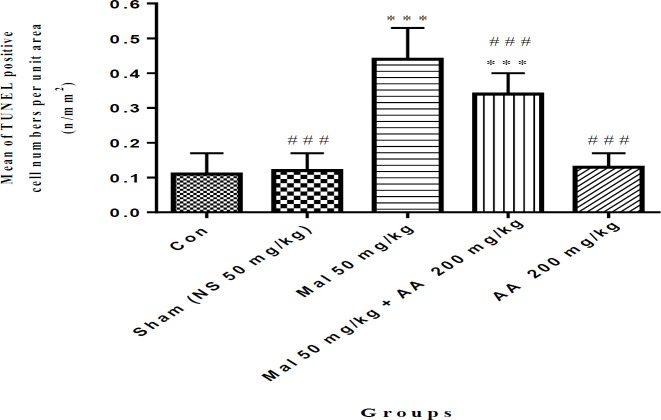
Comparison of the number of apoptotic cells in primary spermatocyte cells among different groups, data are shown as mean±SD, One-way ANOVA, and Tukey-Kramer. Mal 50 mg/kg and AA 200 mg/kg were administered IP once a day and seven times per week. ***P<0.001 compared with the Con group, and ###*P*<0.001 compared with Mal group

In this study, malathion 50 mg/kg significantly increased the number of apoptosis cells in the primary spermatocyte cells compared with the control group (*P*<0.001, whereas co-administration of malathion 50 mg/kg and ascorbic acid 200 mg/kg decreased the number of apoptotic cells compared with malathion 50 mg/kg group (*P*<0.001). Ascorbic acid 200 mg/kg also significantly reduced the number of apoptotic cells in primary spermatocyte cells compared with the malathion 50 mg/kg group (*P*<0.001) (Figures 3 and 4). In addition, the number of apoptotic cells in primary spermatocyte cells was not significantly different in sham, ascorbic acid, and control groups (*P*>0.05).

***Effect of ascorbic acid on proliferation of spermatogonia cells after malathion exposure***

**Figure 5:**
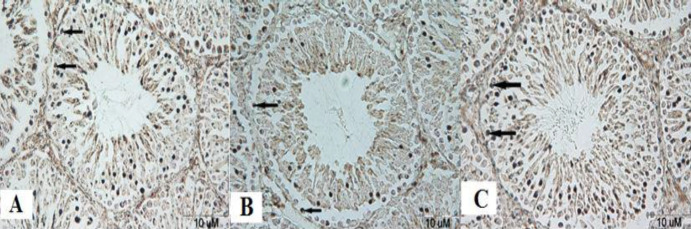
The microscopic sections of proliferation of spermatogonia cells in male rat testis by PCNA technique. Sections were cut into 5 µm thickness and stained with Hematoxylin. The PCNA-positive nuclei are seen in brown. The spermatogonia cells are indicated by arrows. Control group, no PCNA-positive cells, magnification ×40 (A). In malathion 50 mg/kg group the number of spermatogonia cells significantly decreased compared to control group, magnification ×40 (B), and in malathion 50 mg/kg along with ascorbic acid 200 mg/kg the number of spermatogonia cells significantly increased compared with the malathion 50 mg/kg group, magnification ×40 (C)

**Figure 6:**
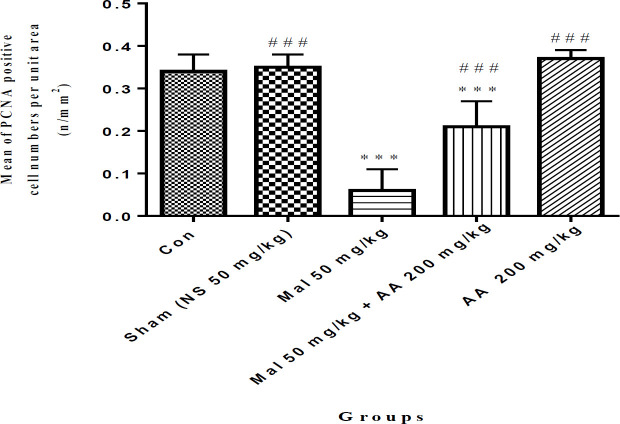
Comparison of the proliferation of spermatogonia cells among different groups, data are shown as mean±SD, One-way ANOVA, and Tukey-Kramer. Mal 50 mg/kg and AA 200 mg/kg were administered IP once a day and seven times per week. ***P<0.001 compared with the Con group, and ###*P*<0.001 compared with Mal group

In this study, the PCNA technique showed that the number of proliferation of spermatogonia cells was significantly decreased in malathion 50 mg/kg compared with the control group (*P*<0.001). While malathion 50 mg/kg along with ascorbic acid 200 mg/kg increased proliferation of spermatogonia cells compared with the malathion 50 mg/kg group (*P*<0.001). Ascorbic acid 200 mg/kg also significantly increased the proliferation of spermatogonia cells compared with malathion 50 mg/kg (*P*<0.001) (Figures 5 and 6). In addition, the proliferation of spermatogonia cells was not significantly different in sham, ascorbic acid, and Con groups (*P*>0.05).

***Effect of ascorbic acid on proliferation of primary spermatocyte cells after malathion exposure***

**Figure 7:**
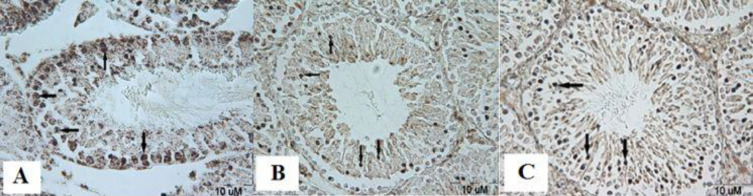
The microscopic sections of the proliferation of primary spermatocyte cells in male rat testis by the PCNA technique. Sections were cut into 5 µm thickness and stained with Hematoxylin. The PCNA-positive nuclei are seen in brown. The primary spermatogonia cells are indicated by arrows. Control group, no PCNA-positive cells, magnification ×40 (A). In malathion 50 mg/kg group the number of the primary spermatocyte cells significantly decreased compared with the control group, magnification ×40 (B), and in malathion 50 mg/kg along with ascorbic acid 200 mg/kg group the number of the primary spermatocyte cells significantly increased compared to the malathion 50 mg/kg group, magnification ×40 (C)

**Figure 8:**
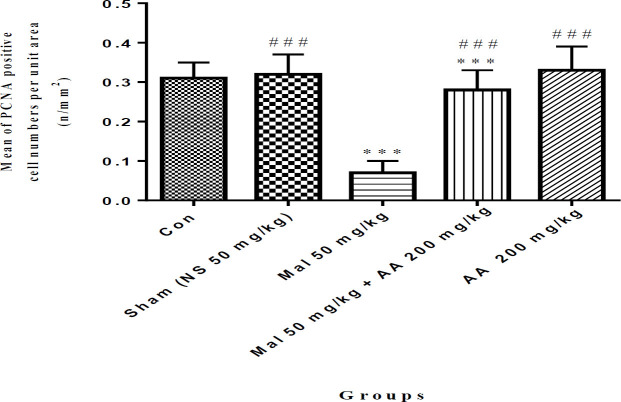
Comparison of the proliferation of primary spermatocyte cells among different groups, data are shown as mean±SD, One-way ANOVA, and Tukey-Kramer. Mal 50 mg/kg and AA 200 mg/kg were administered IP once a day and seven times per week. ***P<0.001 compared with the Con group, and ###*P*<0.001 compared with Mal group

As shown in Figures 7 and 8, the proliferation number of primary spermatocyte cells was significantly decreased in malathion 50 mg/kg compared with the control group (*P*<0.001). Malathion 50 mg/kg, along with ascorbic acid 200 mg/kg increased the proliferation of primary spermatocyte cells compared with malathion 50 mg/kg (*P*<0.001). Ascorbic acid 200 mg/kg also significantly increased the proliferation of spermatogonia cells compared with malathion 50 mg/kg (*P*<0.001) (Figures 7 and 8). In addition, the proliferation of primary spermatocyte cells was not significantly different in sham, ascorbic acid, and control groups (*P*> 0.05). 

## Discussion

In this study, the protective effects of ascorbic acid on malathion-induced toxicity in adult male rat testis were investigated. The effect of malathion on apoptosis and proliferation of spermatogonia and primary spermatocyte cells was also evaluated by TUNEL and PCNA techniques, respectively. 

For the first time in this study, we proved that malathion at a dose of 50 mg/kg increased apoptosis of spermatogonia and primary spermatocyte cells in adult male Wistar Rats while ascorbic acid significantly reduced the number of apoptotic cells. The proliferation of spermatogonia and primary spermatocyte cells was significantly reduced in the malathion group, while co-administration of malathion and ascorbic acid significantly increased the number of these cells. Also, the proliferation of cells in the control, normal saline, and ascorbic acid groups was not significantly different.

OP pesticides increased apoptosis by inducing oxidative stress, releasing cytochrome C from mitochondria and activating caspases 3 and 9, leading to activation of internal and external pathways of apoptosis (23) in various tissues of the body, such as the gonads (43). Another study proved that malathion induced apoptosis in spermatogonia types A and B, spermatocyte, and spermatid cells, and decreased activity of acetylcholine esterase enzymes in mice testis (43). Malathion at a dose of 241 mg/kg also increased apoptosis of germ cells in the seminiferous tubules and decreased activity of the cholinesterase plasma enzyme (44).

In previous studies, it was found that ascorbic acid can improve cell antioxidant defense and protect the cell against oxidative stress-induced apoptosis. For example, it was shown that apoptosis induced by OP pesticides such as diclorrosis, methyl parathion, and chlorpyrifos in endometrial tissue (13, 45) and retinal cells (46) can be reduced with vitamins C and E. The similar effects of vitamins E and crocin were shown on apoptotic effect of diazinon (an organophosphate) and atrazine (a herbicide) in other studies. It was revealed that diazinon and atrazine significantly increased the number of apoptotic cells detected via the TUNEL staining technique in the ovary of adult female Wistar rat and Balb/c mice testis, respectively (38, 47). In the current study, MDMA (3,4-methylenedioxymethamphetamine, ecstasy) significantly increased the number of apoptotic cells detected via the TUNEL staining technique in adult rat testis (39). In addition, another study demonstrated that ascorbic acid reduced endosulfan-induced cardiotoxicity in rabbits (37). 

A previous study observed that DNA replication is a sign of proliferation and division of cells and conversion of phase G_1 _to S (20). PCNA expression is directly related to mitotic activity, so it can be considered to be a marker of cell proliferation for body cells such as spermatogonia and primary spermatocytes cells in testis tissue (48). OP pesticides, such as malathion, have led to DNA damage, prevented the cell cycle, and induced apoptosis (49) in testis tissue through the formation of free radicals and oxidative stress (50).

Histological findings also confirmed that exposure of testis to malathion has many different complications. In other words, OP pesticides, especially malathion, are the most commonly used pesticides in rice fields and citrus gardens (2). Therefore, malathion poisoning creates hormonal disorders of the endocrine system and infertility (2). 

The limitations of this study were not evaluating inflammatory factors and internal and external pathways of apoptosis. It is also recommended that in future studies, the effects of other antioxidants such as crocin, curcumin, etc. as protective agents could be used.

Con: Control; Mal: Malathion; AA: Ascorbic acid; NS: Normal saline; IP: Intraperitoneal

Con: Control; Mal: Malathion; AA: Ascorbic acid; NS: Normal saline; IP: Intraperitoneal

Con: Control; Mal: Malathion; AA: Ascorbic acid; NS: Normal saline; IP: Intraperitoneal

Con: Control; Mal: Malathion; AA: Ascorbic acid, NS: Normal saline; IP: Intraperitoneal

## Conclusion

In the present study, we revealed that the use of an appropriate antioxidant such as ascorbic acid reduces the side effects of malathion. Also, generalization of the results of this study to human populations exposed to direct (inhalation or contact) or indirect (consumption of agricultural and contaminated protein substances) organophosphorus pesticides can be a serious environmental pollution warning. Therefore, it is suggested that suitable antioxidants such as ascorbic acid be used as treatments for organophosphorus pesticide-induced poisoning.
